# Immune responses of patients on maintenance hemodialysis after infection by SARS-CoV-2: a prospective observational cohort study

**DOI:** 10.1186/s12879-023-08569-2

**Published:** 2023-09-06

**Authors:** Dimitra Bacharaki, Minas Karagiannis, Panagiotis Giannakopoulos, Evangelos Papachristou, Dimitrios Divanis, Aggeliki Sardeli, Dimitra Petrou, Petros Nikolopoulos, Adamantia Bratsiakou, Vassiliki Zoi, Nikitas Piliouras, Georgia Damoraki, Vassilios Liakopoulos, Dimitrios Goumenos, Evangelos J. Giamarellos-Bourboulis

**Affiliations:** 1 Department of Nephrology, University General Hospital Attikon, Athens, Greece; 2 Department of Nephrology, Rion University Hospital, University of Patras, Patras, Greece; 3 2nd Department of Nephrology, AHEPA Hospital, Medical School, Aristotle University of Thessaloniki, Thessaloniki, Greece; 4 4th Department of Internal Medicine, National and Kapodistrian University of Athens, Athens, Greece

**Keywords:** COVID-19, Hemodialysis, Cytokines, HLA-DR, Immune response

## Abstract

**Background:**

Immune dysregulation in patients with acute COVID-19 under chronic hemodialysis (CHD) is fully not elucidated. The changes of mononuclear counts and mediators before and after HD and associations with final outcome were studied.

**Method:**

In this prospective study, hospitalized patients with moderate-to-severe COVID-19 under CHD and matched comparators under HD were analyzed for their absolute counts of lymphoid cells and circulating inflammatory mediators. Blood samples were collected before start and at the end of the first HD session; dialysate samples were also collected.

**Result:**

Fifty-nine patients with acute COVID-19 under CHD and 20 uninfected comparators under CHD were enrolled. Circulating concentrations of tumor necrosis factor-alpha (TNFα), interleukin (IL)-10, interferon-γ and platelet-derived growth factor-A were increased in patients. Concentrations of mediators did not differ before and after HD. Significant decreases of CD4-lymphocytes and CD19-lymphocytes were found in patients. The decrease of the expression of HLA-DR on CD14-monocytes was associated with unfavorable outcome (defined as WHO-CPS 6 or more by day 28); increased counts of CD19-lymphocytes were associated with better outcomes.

**Conclusion:**

Patients under CHD develop an inflammatory reaction to SARS-CoV-2 characterized by increase of inflammatory mediators, decrease of circulating T-lymphocytes and decrease of the expression of HLA-DR on CD14-monocytes.

## Introduction

The pandemic of Coronavirus Disease 19 (COVID-19) from the new Severe Acute Respiratory Syndrome Coronavirus 2 (SARS-CoV-2), originating from China on December 2019, is still menacing with huge social and financial consequences [1]. When severe respiratory failure becomes apparent, patients present with a complex immune dysregulation presenting features of both hyper-immune activation and immunoparalysis [2, 3]. Elderly people and patients with comorbidities like type 2 diabetes mellitus, chronic obstructive pulmonary disease, coronary artery disease, obesity and chronic kidney disease (CKD) are considered populations at risk for unfavorable outcome [4, 5]. The risk is more prevailing among patents at End-Stage Renal Disease (ESRD) on chronic hemodialysis (CHD) [6]. CHD in an independent state of immune paralysis and immune activation [7, 8] often described as “inflammaging” and this may explain the vulnerability to unfavorable outcome.

Patients at CHD present with delayed clearance of the virus and a defective response to vaccination [9]. However, there are still several aspects of the immune response to SARS-CoV-2 in CHD which remain unclear: a) which are the cytokines and white blood subsets which are implicated in the immune response; b) can CHD modulate inflammatory mediators so as to offer protection from an exacerbated inflammatory reaction; and c) can the early immune responses define outcomes? In order to provide replies to these questions we used a study design which has not been applied so far; patients with COVID-19 on stable CHD were compared to matched comparators on CHD. Patients with severe COVID-19 already in need of mechanical ventilation (MV) or non-invasive ventilation (NIV) were excluded in order to decipher the role of early immune responses to 28-day outcomes.

## Patients and methods

### Study design

This is an observational prospective cohort study conducted in the Hemodialysis Units of three University Hospitals in Greece (Attikon University General Hospital, University General Hospital of Patras and AHEPA University General Hospital of Thessaloniki) between March 2021 and February 2022. The study protocol and informed consent form were approved by the Institutional Review Boards (approvals 48/24.02.2021; 161/01.04.2021 and 199/30.3.2021 respectively).

Inclusion criteria were: 1) age 18 years or more; 2) estimated glomerular filtration rate (eGFR) less than 15 ml/min/1.73m^2^estimated by the CKD Epidemiology Collaboration (CKD-EPI) creatinine Eq [10]. and at least three consecutive months on CHD; 3) infection by SARS-CoV-2 defined by positive RT-PCR of the nasopharyngeal swab; and d) hospitalization without need of MV or NIV. During the study period, all CHD patients infected by SARS-CoV-2 were hospitalized in public hospitals irrespective of COVID-19 severity since they were considered high risk patients. Hospitalization at private hospitals was not allowed.

Exclusion criteria were: 1) age less than 18 years; 2) infection by the human immunodeficiency virus; 3) absolute neutrophil count less than 1,000/mm^3^; 4) treatment with biological agents targeting cytokines or cell receptors during the last one month; 5) chronic intake of corticosteroids defined as oral or intravenous daily intake of prednisolone (or equivalent) dose 0.4 mg/Kg the last month. This one-month period is considered in most randomized clinical trials adequate wash out from the immunomodulating effect of any previous corticosteroid or biological treatment.

One comparator group was enrolled using the following criteria of matching: 1) age and gender; 2) at least 3 months on CHD; and 3) Charlson’s Comorbidity Index (CCI). Comparators were not infected by SARS-CoV-2 as defined by negative RT-PCR of one nasopharyngeal swab. The same exclusion criteria applied for comparators as for patients with COVID-19.

The following information was recorded: demographics; reason of primary renal disease; CCI; COVID-19 severity according to WHO; frailty index [11]; history of vaccination and number of vaccine doses; white blood cell count, C-reactive protein, procalcitonin, ferritin, urea and creatinine on hospital admission; type of administered therapy; and WHO-CPS (World Health Organization Clinical Progression Scale) at days 14 and 28 [12]. The WHO-CPS is an ordinal score ranging from 0 (fully recovered) to 10 (dead) reflecting the different stages of severity of COVID-19. For patients who were discharged before day 28, the WHO-CPS was captured after phone contact.

All patients were receiving standard-of-care treatment according to the WHO guidance including supplementary oxygen to maintain oxygen saturation above 93%; intravenous 6 mg dexamethasone once daily for 10 days or until discharge [13] and low molecular weight heparin. Intake of remdesivir and antibiotics was at the discretion of the attending physicians. HD sessions were performed in an isolated room of the study hospital, regularly three times per week, or more as per patient needs. HD was done using low or high flux filters at the discretion of the attending physician and recorded. Low and high flux filters were discriminated by predefined characteristics as follows: a) ultrafiltration coefficient greater or less than 12 mL/h/mm for high and low flux respectively; or b) beta‐2 microglobulin clearance greater than 20 mL/min or less than 10 mL/min for high and low flux respectively.

Blood was collected on the first time of HD session in the study hospital and always less than 48 h after hospital admission. Eight ml of peripheral whole blood was collected immediately before the start of HD under sterile conditions. Another eight ml was collected at the end of HD. From the collected samples, five ml was poured into sterile tubes (Vacutainer, BD) and used for cytokine measurements; and another three ml was poured into one EDTA-coated tube (Vacutainer BD) and used for flow cytometry. Five ml of the HD effluent dialysate was collected 30 min after the start of the HD session into one sterile tube (Vacutainer, BD) and used for cytokine measurements.

### Laboratory investigation

Blood was centrifuged and serum was stored in -80 °C until assayed. Levels of biomarkers were quantified by using commercially available kits of enzyme immunosorbent assays from Bio-Techne (R&D Systems, Minneapolis, MN) according to the manufacturer’s instructions. The lower limits of detection were: 16 pg/ml for tumor necrosis factor-alpha (TNFα); 40 pg/ml for IL-6; 31 pg/ml for IL-10; 62 pg/ml for IL-38; 156 pg/ml for IFNγ; and 313 p/ml for platelet-derived growth factor (PDGF)-A.

White blood cells were incubated for 15 minutes in the dark with the monoclonal antibodies anti-CD14 FITC, anti-HLA-DR-PE, anti-CD45 PC5 (Beckman Coulter, Marseille, France). White blood cells were also incubated for 15 minutes in the dark with anti-CD3 FITC, anti-CD4 FITC and anti-CD19 FITC (fluorescein isothiocyanate, emission 525nm, Beckman Coulter); with anti-CD4 PE, anti-CD8 PE, and anti-CD(16+56) PE (phycoerythrin, emission 575nm, Beckman Coulter); and with anti-CD45 PC5 (emission 667nm, Beckman Coulter). Fluorospheres (Beckman Coulter) were used for the determination of absolute counts. Cells were analyzed after running through the CYTOMICS FC500 flow cytometer (Beckman Coulter Co, Miami, Florida). Isotypic IgG controls stained also with anti-CD45 were used for each patient. Results of HLA-DR on CD14/CD45-cells were expressed as mean fluorescence intensity (MFI).

### Study endpoints

The primary study endpoint were the differences between cytokines and white blood cell subsets between patients on CHD and COVID-19 and comparators on CHD.

The secondary study endpoint was to identify the best predictor of the 28-day outcome. Pre-defined outcomes were WHO-CPS of 6 or more (interpreted as hospitalized with severe disease or dead) at day 28 and WHO-CPS 3 or less at day 28 (interpreted as ambulatory disease).

### Statistical analysis

The calculation of the study power was done with the assumption that at least two of the measured variables would differ statistically between patients and comparators at a p value less than 0.0001 and that the true difference would be 150 with 210 standard deviation. To demonstrate this difference with 80% power at the 10% level of significance with patient: comparator ratio 3:1, 54 experimental subjects and 16 control subjects were needed.

Results are presented as medians and distribution. Comparisons between patients and comparators were done by the Mann Whitney U test. Paired comparisons before HD and at the end of HD were done by the Wilcoxon ranked sum test. Patients were split into subgroups based on their outcome at day 28. The subgroups were: those with WHO-CPS ≥ 6 and WHO-CPS < 6; and those with WHO-CPS ≤ 3 and WHO-CPS > 3. Comparison of cytokines and white blood cell subsets between subgroups were done by the Mann–Whitney U test. The cytokine or cell subset for which significant differences were found was analyzed through ROC (receiver operator characteristics) curve to define a cut-off which defines outcome. The cut-off was found after applying the Youden index. The sensitivity, specificity, positive predictive value (PPV) and negative predictive value (NPV) of the cut-off for the outcome was calculated; the odds ratio (OR) and 95% confidence intervals were defined by the Mantel–Haenszel statistics. Any value of p less than 0.05 was considered significant.

## Results

### Study population

**Fig. 1:**
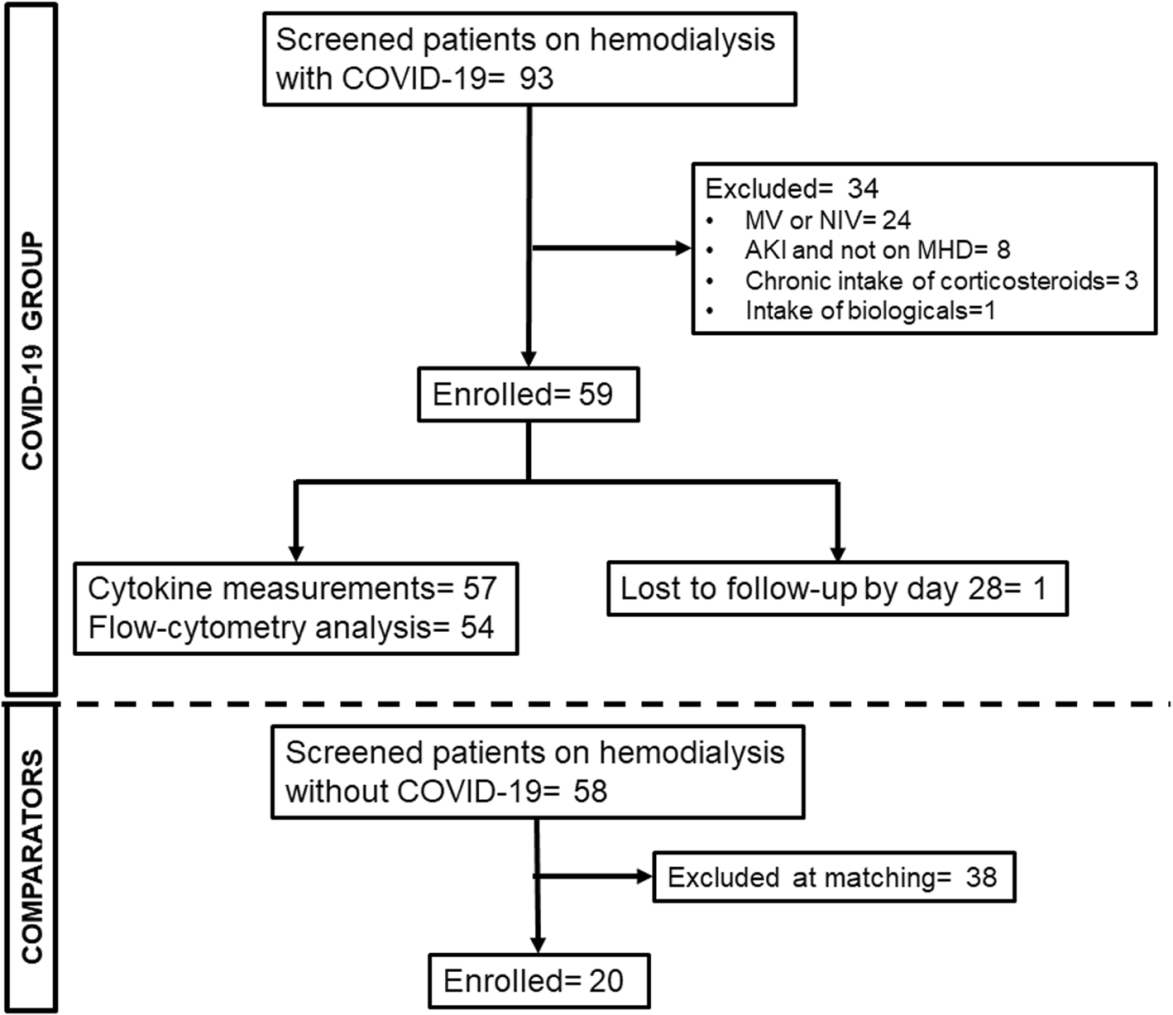
Study flow-chart. Abbreviations: AKI, acute kidney injury; COVID, coronavirus disease; CHD, maintenance hemodialysis; MV, mechanical ventilation; NIV, non-invasive ventilation

The first patient was enrolled on 17 March 2021 and the follow-up of the last patient was completed on 30 April 2022. The study flow chart is shown in Fig. 1. Fifty-nine patients and 20 comparators were enrolled. The two groups were well-matched for age, gender, comorbidities and frailty index (Table 1). Three (5.1%) and nil (0%) patients, respectively, died by day 28.

**Table 1:** Demographics of enrolled patients and matched comparators

	**Patients**	**Comparators**	***p*-value**
Age, years, mean (SD)	68.0 (16.3)	62.8 (12.7)	0.200
Male gender, n (%)	32 (55.2)	9 (45.0)	0.450
Dialysis vintage, months, median (range)	33.5 (1–320)	66.5 (5–335)	0.168
Charlson’s comorbidity Index, mean (SD)	4.82 (1.85)	5.75 (2.53)	0.085
Frailty score, mean (SD)	3.65 (2.19)	3.35 (1.53)	0.568
Anti-SARS-CoV-2 vaccination, n (%)	44 (74.6)	18 (90.0)	0.212
Number of vaccine doses, mean (SD)	1.52 (1.07)	2.65 (1.08)	< 0.0001
Moderate COVID-19, n (%)	59 (100)	NA	
Absolute total white blood cell count/mm^3^, mean (SD)	6625.2 (2760.6)	7204.6 (2312.5)	0.407
Absolute neutrophil blood cell count/mm^3^, mean (SD)	4869.1 (2562.7)	5321.4 (2562.7)	0.485
Absolute lymphocyte blood cell count/mm^3^, mean (SD)	988.1 (446.7)	1371.2 (673.2)	0.006
C-reactive protein, mg/l, median (range)	18.0 (0.4–261.7)	3.1 (0.5–28.0)	< 0.0001
Procalcitonin, ng/ml, median (range)	0.82 (0.14–30.40)	0.23 (0.07–0.39)	< 0.0001
Ferritin, ng/ml, median (range)	720 (27–7430)	42.5 (5.9–374.3)	< 0.0001
Urea, mg/dl mean (SD)	132.9 (45.4)	130.4 (30.9)	0.827
Creatinine, mg/dl mean (SD)	7.34 (2.56)	8.47 (1.79)	0.087
Medical history of diabetes mellitus, n (%)	27 (46.6)	8 (40.0)	0.795
Medical history of arterial hypertension, n (%)	46 (80.7)	11 (55.0)	0.037
Obesity, n (%)	12 (20.7)	5 (25.0)	0.756

*Abbreviations*: *n* Number of patients, *NA* Non-available, *SD* Standard deviation

### Primary endpoint: circulating mediators and white blood cell subsets

**Fig. 2:**
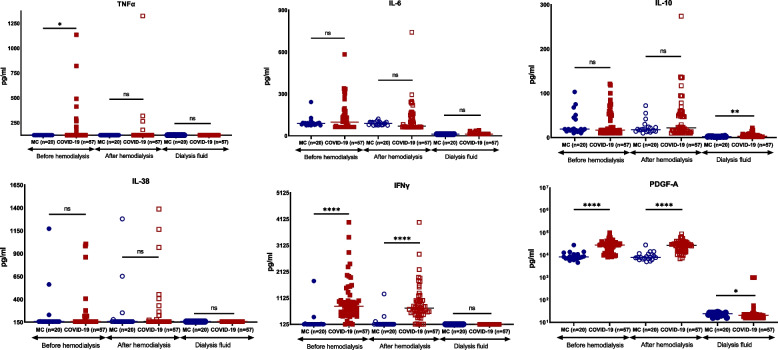
Comparative concentrations of circulating cytokines. Circulating cytokines were measured before start of hemodialysis, after the end of hemodialysis and in the dialysate of patients on maintenance hemodialysis with COVID-19 and matched comparators. Line represents the median of the distribution. Comparisons between patients and comparators are shown: ns, non-significant; *p < 0.05; ****p < 0.0001. Abbreviations: IFN, interferon; IL, interleukin; MC, matched comparators; n, number of patients; PDGF, platelet-derived growth factor; TNF, tumour necrosis factor

Among circulating cytokines, TNFα, IFNγ and PDGF-A were higher in patients than comparators (Fig. 2). These differences were found on both times of sampling i.e. before start of HD and after HD. The only exception was TNFα which was higher in patients only before start of HD. The concentrations of cytokines found in the dialysate were low with the exceptions of IL-10 and PDGF-A. Both IL-10 and PDGF-A were increased in the dialysate of COVID-19 patients.

**Fig. 3:**
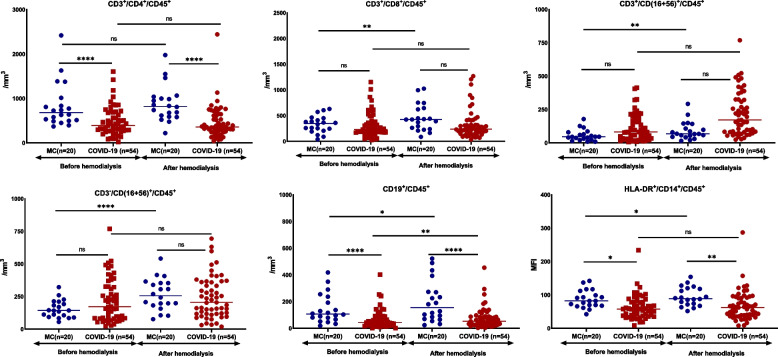
Comparative white blood cell subsets. Absolute counts of lymphocyte subsets were measured before start of hemodialysis and at the end of hemodialysis in patients on maintenance hemodialysis with COVID-19 and matched comparators. Line represents the median of the distribution. Comparisons are shown: ns, non-significant; *p < 0.05; ****p < 0.0001. Abbreviations: MC, matched comparators; MFI, mean fluorescence intensity; n, number of patients

The absolute counts of CD4-lymphocytes and of CD19-lymphocytes were significantly lower in patients than comparators both before and after the HD session. The expression of HLA-DR on CD14-monocytes of patients, provided as the MFI, was significantly lower than comparators both before and after the HD session (Fig. 3).

### Predictors of outcome

**Fig. 4:**
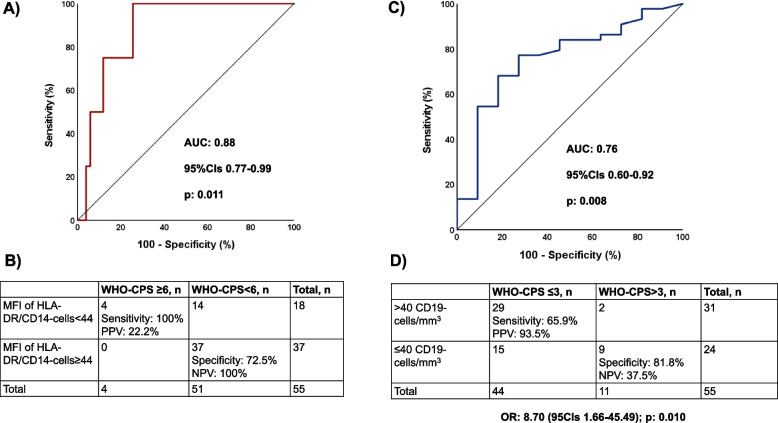
Baseline predictor of 28-day outcome. **A** ROC curve of MFI of HLA-DR on CD14-monocytes at the end of hemodialysis to predict WHO-CPS ≥ 6 at day 28. **B** Sensitivity, specificity, PPV and NPV of MFI < 44 HLA-DR on CD14-monocytes at the end of hemodialysis to predict WHO-CPS ≥ 6 at day 28. The OR could not be calculated because two values were zero. **C** ROC curve of the CD19-cell count at the end of hemodialysis to predict WHO-CPS ≤ 3 at day 28. **D** Sensitivity, specificity, PPV and NPV of CD19-cell count more than 40/mm^3^ at the end of hemodialysis to predict WHO-CPS ≤ 3 at day 28. The OR is provided. Abbreviations: AUC, area under the curve; CI, confidence interval; CPS, clinical performance scale; MFI, mean fluorescence intensity; n, number of patients; OR, odds ratio; NPV, negative predictive value; PPV, positive predictive value; ROC, receiver operator characteristics; WHO, World Health Organization

When baseline demographics, circulating cytokines and cell subsets were compared between patients who at day 28 were at WHO-CPS ≥ 6 and at WHO-CPS < 6, no differences were found (data not shown) with the only exception of the expression of HLA-DR on CD14-monocytes at the end of HD which was lower among patients with WHO-CPS ≥ 6. ROC curve analysis showed that MFI less than 44 was an independent predictor of outcome with 100% sensitivity and 100% NPV (Fig. 4A and B).

When baseline demographics, circulating cytokines and cell subsets were compared between patients who at day 28 were at WHO-CPS ≤ 3 and at WHO-CPS > 3, no differences were found (data not shown) with the only exception of the absolute counts of CD19-lymphocytes at the end of HD which were greater among patients with WHO-CPS ≤ 3. ROC curve analysis showed that an absolute CD19-cell count more than 40/mm^3^ was an independent predictor of outcome with 93.5%PPV and 81.8% specificity (Fig. 4C and D). The OR for WHO-CPS at day 28 3 or less was 8.70 when the baseline CD19-cell count was more than 40/mm^3^.

## Discussion

The current study showed that patients under CHD hospitalized for moderate COVID-19 have increased circulating levels of TNFα, IFNγ and PDGF-A, decreased CD14-lymphocytes, decreased CD19-lymphocytes and decreased expression of HLA-DR on CD14-monocytes. The filter of HD did not change the circulating concentrations of the measured mediators. The main predictors of 28-day outcome, as this is expressed by the WHO-CPS, were CD19-subsets and the expression of HLA-DR. More precisely, patients with defective expression of HLA-DR on CD14-monocytes were prone to unfavorable outcome with WHO-CPS 6 or more i.e. severe disease or dead. On the contrary, patients with more than 40 CD19-lymphocytes/mm^3^ were at greater likelihood for favorable outcomes with WHO-CPS 3 or less i.e. discharge home with or without symptoms.

Existing observational studies report increased mortality of patients under CHD from COVID-19 [14, 15], although some studies suggest that dialysis patients are rather protected from the severe form of COVID-19 [16] probably because they are unable to develop pro-inflammatory responses similar to the non-CHD patients [17]. Our results are in general agreement with the results of another study of 32 patients with COVID-19 on CHD [18]. However, that study did not report on the expression of HLA-DR on CD14-monocytes whereas no measurements were done before and at the end of the HD session.

Patients at CHD present immmunosenescence expresse through lymphopenia and low-grade chronic inflammation [18]. Indeed, following HD CD8-lymphocytes, NK-cells, NKT-cells, CD19-lymphocytes and the expression of HLA-DR on CD14-monocytes were increased in the comparator group. Our findings suggest that when patients are acutely infected by SARS-CoV-2 they present further attenuation of T-cell and B-cell responses and defective HLA-DR presentation on monocytes. In these patients the HD filter could not absorb circulating cytokines. This probably implies that the lack of retention of cytokines by the HD filter does not influence outcome. However, controversial findings are published on the retention of circulating cytokines by the HD filter in COVID-19. In an observational study of 60 patients, there were no differences regarding the need for admission in the intensive care unit and the incidence of death between patients under low-flux HD and patients under medium flux HD [19]. Special emphasis should be given to three studies. In the first study 74 patients under CHD infected by SARS-CoV-2 were enrolled. The use of filters by asymmetric cellulose triacetate (ATA) was compared to filters by polymethylmethacrylate (PMMA). Using a multivariate logistic regression model, the authors concluded that ATA favoured the retention of IL-6 [20]. In the second small-scale randomized trial, 15 patients underwent HD using medium cut-off (MCO) membranes and were compared to 14 patients who underwent HD using filters by PMMA. Results showed that following the session of HD using MCO, circulating IL-8 and IL-10 decreased. IL-8 was also decreased after the HD session using PMMA [21]. In the third study, nine patients under CHD with COVID-19 were subject to HD, using MCO filter. IL-6 levels before and after dialysis were not uniform in all patients. However, IL-6 was increased between serial HD session among non-survivors and overall circulating IL-6 was higher in non-survivors [22].

One fully novel finding was the increase of IL-10 in the effluent dialysate of HD. This can be partly explained by the greater anti-inflammatory burden in patients under CHD. Although considered a major anti- inflammatory cytokine, early rise of IL-10 is associated and possibly involved in severe COVID-19 [23]. Similar increases were shown for PDGF-A levels. Increased levels of PDGF-A have been described in moderate COVID-19 and showed inverse correlation with circulating D-dimers [24]. PDGF-A is a marker of endothelial dysfunction and COVID-19 is a state of endotheliitis [25]. The overall favourable outcome of the patients generates the question if this increase is protective from immune exhaustion.

Despite the enthusiasm generated at the early stages of the pandemic that COVID-19 is an IL-6-driven disease, it soon became evident that not all severe patients have increased IL-6 at hospital admission [17, 26]. This seems to be the case of patients under CHD. Another feature of the immune response in these patients is the decrease of the expression of HLA-DR on monocytes. This denotes defect of antigen-presentation which contributes to the immune dysregulation of COVID-19 hallmarked by B-cell lymphopenia and defective antibody production. On the contrary, increased B-lymphocyte counts in infected patients under CHD are associated with better outcomes.

The major strengths of the present study are the participation of patients from three different departments and the study design providing cytokine measurements before and at the end of the HD session and from the dialysate. Several limitations of the study need to be acknowledged. These limitations are: i) the small number of patients; ii) the lack of serial day measurements; iii) the inclusion of comparators vaccinated with more vaccine doses than patients. Medical history of vaccination may induce false positive increase of cytokine levels and white blood cell subpopulations. Since vaccine dosing was more intense in the comparators than the patients, this false positive effect if limited; iv) the lack of ferritin data of patients before they were infected with SARS-CoV-2. This does not allow to fully address COVID-19 as the single reason of hyper-ferritinemia. However, increase of ferritin is so common among non-CHD patients [2, 3] that SARS-CoV-2 infection may be considered the reason of ferritin increase with relative certainty; and v) the lack of information on viral variants.

## Conclusion

Our findings suggest that patients under CHD develop an inflammatory reaction to SARS-CoV-2 which is characterized by increase of inflammatory mediators, decrease of circulating T-lymphocytes and decrease of the expression of HLA-DR on CD14-monocytes. The expression of HLA-DR on circulating monocytes and the CD19-counts are determinants of the final disposition.

## Data Availability

Data are available from the corresponding author upon request.
